# Circulating Beta-Defensin 2 Levels Correlate with Conventional Inflammatory Markers in Infection-Free Individuals with Overweight and Obesity: An Exploratory Study

**DOI:** 10.3390/biomedicines13081800

**Published:** 2025-07-23

**Authors:** Theocharis Koufakis, Dimitrios Kouroupis, Georgios Dimakopoulos, Theofylaktos Georgiadis, Areti Kourti, Paraskevi Karalazou, Katerina Thisiadou, Panagiotis Doukelis, Ioanna Zografou, Dimitrios Patoulias, Djordje S. Popovic, Athina Pyrpasopoulou, Evangelos Fousteris, Georgia Argyrakopoulou, Alexander Kokkinos, Olga Giouleme, Kalliopi Kotsa, Michael Doumas, Kali Makedou

**Affiliations:** 1 Second Propaedeutic Department of Internal Medicine, Hippokration General Hospital, Aristotle University of Thessaloniki, 54642 Thessaloniki, Greece; dimcour841@gmail.com (D.K.); pitdukel@yahoo.gr (P.D.); ioannazo@yahoo.gr (I.Z.); dipatoulias@gmail.com (D.P.); a.pyrpasopoulou@doctors.org.uk (A.P.); olga.giouleme@gmail.com (O.G.); michalisdoumas@yahoo.co.uk (M.D.); 2 BIOSTATS, Epirus Science and Technology Park Campus, University of Ioannina, 45110 Ioannina, Greece; info@biostats.gr; 3 National Primary Health Care Network, 61200 Polykastron, Greece; gfiltheo@hotmail.com; 4 Laboratory of Biochemistry, AHEPA University Hospital, School of Medicine, Aristotle University of Thessaloniki, 54124 Thessaloniki, Greece; aretikourti@auth.gr (A.K.); vivikarala@gmail.com (P.K.); thisiadou@yahoo.gr (K.T.); kalimakedou@gmail.com (K.M.); 5 Clinic for Endocrinology, Diabetes and Metabolic Disorders, Clinical Centre of Vojvodina, Medical Faculty, University of Novi Sad, 21000 Novi Sad, Serbia; pitstop021@gmail.com; 6 Mediterranean Diabetes and Obesity Clinics, 17561 Athens, Greece; vangelis.fousteris@gmail.com; 7 Diabetes and Obesity Unit, Athens Medical Center, 15125 Athens, Greece; gargyrakopoulou@gmail.com; 8 First Department of Propaedeutic Internal Medicine and Diabetes Center, Laiko General Hospital, Medical School, National and Kapodistrian University of Athens, 11527 Athens, Greece; rjd@otenet.gr; 9 Division of Endocrinology and Metabolism and Diabetes Center, First Department of Internal Medicine, AHEPA University Hospital, Medical School, Aristotle University of Thessaloniki, 54636 Thessaloniki, Greece; kalmanthou@yahoo.gr

**Keywords:** beta defensin 2, obesity, overweight, inflammation, biomarker, intestinal dysbiosis

## Abstract

**Background/Objectives**: The role of intestinal dysbiosis as an important driver of inflammation in metabolic disorders is becoming increasingly evident. Beta-defensin 2 is an antimicrobial peptide that contributes to innate immunity, while recently it has been suggested as a novel biomarker of gut dysbiosis. However, its role in obesity remains unexplored. This study aimed to compare circulating beta-defensin 2 levels between individuals with overweight and obesity and lean controls. An additional objective was to explore potential correlations between beta-defensin 2 and other inflammatory markers in this population. **Methods**: The study population consisted of 81 participants (61.7% females) divided into obesity (n = 27), overweight (n = 34), and normal body mass index (n = 20) groups. All participants were free of infection and diabetes mellitus. Beta-defensin 2, interleukin-6, presepsin, high-sensitivity C-reactive protein (hs-CRP), and ferritin were evaluated in the study groups. **Results**: We did not find significant differences in beta-defensin 2 levels between the groups (*p* = 0.936). In contrast, hs-CRP levels were higher in people with obesity compared to the sum of participants in the overweight and control groups (*p* = 0.044), after adjusting for the effects of age, sex, smoking, and vitamin D status. Furthermore, a positive correlation was established between beta-defensin 2 and presepsin values (*p* = 0.012). **Conclusions**: The results of the present study demonstrate that obesity is characterized by an aggravation of inflammation, as expressed by elevated hs-CRP levels. Although the study design cannot prove causal relationships, our findings also suggest that beta-defensin 2 levels correlate with the magnitude of systemic inflammation in infection-free individuals living with obesity. The value of the combined evaluation of different biomarkers in obesity-related outcomes warrants further investigation by larger studies.

## 1. Introduction

During the past decade, there has been a shift in the perception and management of obesity, from a purely aesthetic disorder to a serious, chronic, and relapsing disease [1]. This change in the perspective of physicians, societies, and healthcare systems is partially attributed to a deeper understanding of the complex underlying pathophysiological mechanisms that undermine weight loss and maintenance in people living with the disease. Obesity is associated with numerous complications, including cardiovascular disorders, chronic kidney disease, obstructive sleep apnea, osteoarthritis, and mental health conditions, and has a deleterious impact on quality of life [2]. Although overweight increases the probability of adverse outcomes compared to the lean state, pathophysiological alterations, such as insulin resistance, lipid abnormalities, and the inflammatory milieu, become more pronounced as the body mass index (BMI) increases, suggesting a close relationship between the risk of complications and the magnitude of adiposity [3].

Obesity is characterized by tissue-specific but also systemic inflammation. There is abundant evidence that circulating levels of inflammatory markers, including high-sensitivity C-reactive protein (hs-CRP), interleukin-6 (IL-6), and ferritin, are significantly higher in people with obesity compared to peers of normal weight [4]. Recently, intestinal dysbiosis has been recognized as an important source of inflammation in metabolic disorders. The high-fat diet and gut bacteria are believed to interact to promote structural changes in the intestinal tract that impair the integrity of its epithelial barrier, leading to increased permeability and antigen leakage into the systemic circulation [5]. This process promotes the generation of immune responses and the production of pro-inflammatory signals that contribute to adipose tissue dysfunction and aggravate insulin resistance [6].

Beta-defensin 2 is an antimicrobial peptide produced by epithelial cells and keratinocytes, which plays an important role in innate immunity as the first line of defense and acts as a potent neutrophil chemoattractant [7]. Although the complete spectrum of beta-defensin 2 functions is still under investigation, it is believed to possess potent anti-infective properties, particularly against infections caused by Gram-negative bacteria and Candida species [8]. It is related to the microbiome through its production in response to microbial contact and its potential impact on the composition and diversity of the gut microbiota. Beta-defensin 2 and other members of the defensin family have been suggested to be important regulators of the barrier function of intestinal epithelial cells, and their levels alter in response to barrier disruption [9]. In support of this notion, beta-defensin 2 has been found to be significantly higher in fecal samples from children with inflammatory bowel disease compared to healthy controls [10]. Furthermore, increased beta-defensin 2 concentrations have been measured in urine samples from patients with ulcerative interstitial cystitis, indicating that this peptide may be a reliable marker of dysbiosis observed in various systems of the human body [11].

Currently, the role of biomarkers in the clinical evaluation of people living with obesity remains limited, highlighting the need to identify new markers that reflect the underlying pathophysiology. In addition, there is a complete knowledge gap on the fluctuation of beta-defensin 2 levels in overweight and obesity. This study aimed to compare circulating beta-defensin 2 concentrations between individuals with overweight and obesity and controls of normal weight, all free of infection and diabetes mellitus. An additional objective was to identify potential correlations between beta-defensin 2 and other inflammatory markers (specifically hs-CRP, presepsin, IL-6, and ferritin) in this population.

## 2. Materials and Methods

### 2.1. Study Design

Conducted between January and May 2024, this cross-sectional study recruited participants living with overweight and obesity from outpatient clinics in Greece. Participants were classified into obesity, overweight, and control (normal weight) groups based on their BMI.

To be eligible, participants needed to meet the following criteria: a. be older than 18 years; b. present a complete medical history at study entry, and c. have a BMI in the range of 18.5–24.9 kg/m^2^ for the control group, 25–29.9 kg/m^2^ for the overweight group, and ≥ 30 kg/m^2^ for the obesity group [12].

The exclusion criteria were defined as follows: (a). presence of recent or ongoing infections, surgical procedures, or inflammatory conditions (e.g., pancreatitis, burns, etc.); (b). a history of diabetes mellitus or prediabetes, according to the standards set by the American Diabetes Association [13]; (c). taking medications that affect body weight, such as corticosteroids or antipsychotics; (d). severe hepatic or renal disease; (e). autoimmune disorders; (f). active cancer or a history of malignant neoplastic disease; and (g). a history of cardiovascular disorders, to mitigate potential confounders related to medications (i.e., acetylsalicylic acid and statins).

### 2.2. Clinical and Laboratory Evaluation

Demographic, anthropometric, and laboratory data were collected for each participant on the same day to minimize seasonal effects on 25-hydroxy-vitamin D [25(OH)D] levels. Demographic data comprised gender, age, and smoking habits. Anthropometric measurements included weight and height. Height was determined using a Holtain wall stadiometer, while weight was measured with a calibrated computerized digital scale (K-Tron P1-SR, Onrion LLC, Bergenfield, NJ, USA) to the nearest 0.1 kg. Participants were assessed barefoot and in light clothing. BMI was computed by dividing the participant’s weight in kilograms by the square of their height in meters. A second investigator independently verified the anthropometric assessment.

Blood samples were collected in the morning following a 12 h overnight fast by antecubital venipuncture, and serum samples were kept at −20 °C until they were analyzed. The 25(OH)D levels were evaluated using the COBAS 8000 (e801 immunochemistry module) with electrochemiluminescence method provided by Roche Diagnostics (Mannheim, Germany). Ferritin and IL-6 were measured with electrochemiluminescence innunoassays on ELECSYS analyzer and hs-CRP with a particle-enhanced immunoturbidimetric assay on a COBAS Pure analyzer provided by the same manufacturer. Beta-defensin 2 levels and presepsin were measured using commercial kits based on sandwich enzyme-linked immunosorbent assay (ELISA) technology (Wuhan Fine Biotech Co, Ltd., Wuhan, China).

### 2.3. Statistical Analysis

Categorical variables were represented by frequencies and percentages, whereas scale measurements were conveyed using means and standard deviations. Following normality tests, medians and ranges were employed to infer statistics across the groups of interest, using the Mann–Whitney test for pairwise comparisons. Spearman’s Rho correlation coefficient was evaluated to assess statistical significance between outcome levels and 25(OH)D and age. Pearson’s chi-square test was used to explore associations between obesity and both vitamin D sufficiency as well as intake. A linear regression model was constructed to identify independent predictors for hs-CRP levels when comparing obese to non-obese groups, incorporating factors such as smoking, age, gender, 25(OH)D levels, and vitamin D intake into the analysis. A significance level of 0.05 was set for all tests, and the analyses were conducted using SPSS version 26.0 (IBM Corp., Armonk, NY, USA).

### 2.4. Ethical Aspects

The research adhered to the principles outlined in the Declaration of Helsinki. All participants provided their informed written consent before entering the study. The research protocol received approval from the Aristotle University of Thessaloniki Bioethics Committee (approval number 192, approved on 5 June 2020).

## 3. Results

### 3.1. Characteristics of the Study Subjects

The study population consisted of 81 individuals, among whom 61.7% were females. The obesity group included 27 subjects, with 34 individuals in the overweight group and 20 participants in the control group. The study subjects had an average age of 47.48 years, an average body weight of 81.63 kg, and an average BMI of 28.81 kg/m^2^. The mean BMIs in the control, overweight, and obesity groups were 22.53, 27.45, and 35.18 kg/m^2^, respectively. One third (33.3%) of the participants were taking vitamin D supplements, which were prescribed by a primary care physician or purchased over the counter. As anticipated, there were significant differences in anthropometric indices between the three groups (*p* < 0.01 for all comparisons). However, there were no substantial differences between the groups in terms of age, gender distribution, and smoking habit, with all *p*-values exceeding 0.05. Additionally, average 25(OH)D levels and duration of supplementation did not differ among the groups (*p* = 0.179 and *p* = 0.324, respectively). Supplementary Table S1 displays the study population’s characteristics sorted by BMI categories.

### 3.2. Inflammatory Markers in the Study Groups

We did not observe significant differences in the values of beta-defensin 2 (*p* = 0.936), presepsin (*p* = 0.175), IL-6 (*p* = 0.341), and ferritin (*p* = 0.237) between the obesity group and the control/overweight group (Table 1).

**Figure 1:**
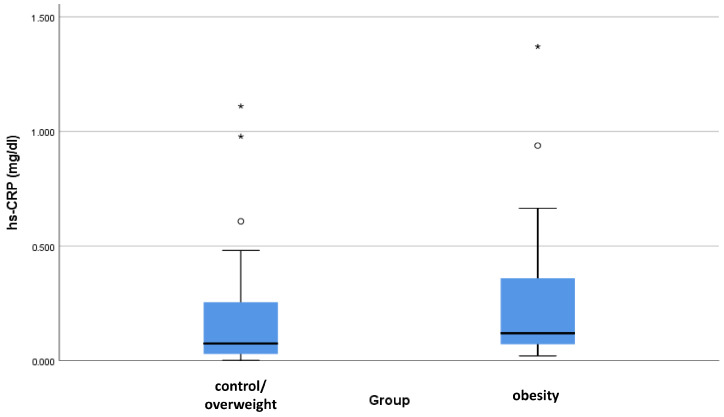
The levels of high-sensitivity C-reactive protein (hs-CRP) in the study groups after adjustment for several confounders. ° Outliers, * Extreme outliers.

The same pattern was observed when the three study groups were examined separately. Hs-CRP was found to be almost twice higher in the obesity group than in the control/overweight group; however, the difference was found to be marginally non-significant (0.36 vs. 0.20 mg/dL, *p* = 0.076). When the analysis was adjusted for age, sex, smoking, 25(OH)D, and vitamin D intake, hs-CRP was found to be significantly higher in the obesity group compared to the comparator group (*p* = 0.044) (Figure 1).

**Figure 2:**
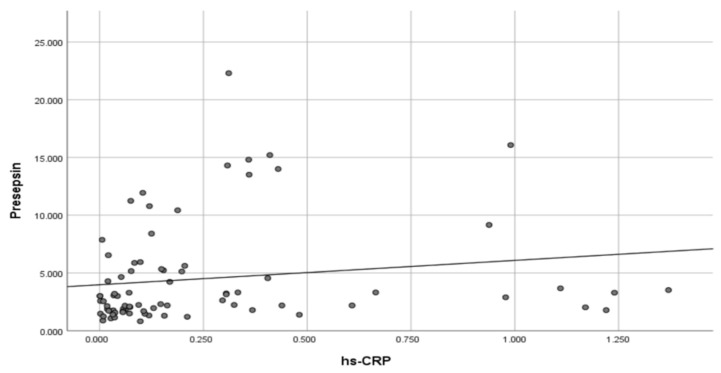
Correlation between presepsin and high-sensitivity C-reactive protein (hs-CRP) values.

We did not find a significant relationship between the hs-CRP values and age (*p* = 0.219), 25(OH)D levels (*p* = 0.871), sex (*p* = 0.777), and vitamin D intake (*p* = 0.186). Statistically significant and positive correlations were found between beta-defensin 2 and presepsin (Rho = 0.283; *p* = 0.012), between presepsin and hs-CRP (Rho = 0.336; *p* = 0.002) (Figure 2), and a marginally non-significant correlation between presepsin and ferritin (Rho = 0.213; *p* = 0.060).

**Figure 3:**
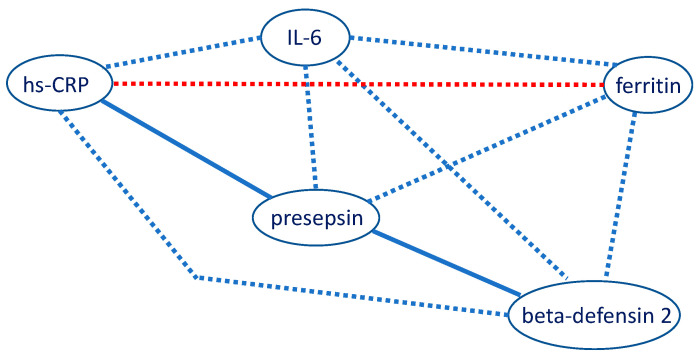
Correlation network of inflammatory markers. Blue = positive correlation. Red = negative correlation. Solid line = statistical significance. Dotted line = no significance. hs-CRP: high-sensitivity C-reactive protein; IL-6: interleukin-6.

There were no significant correlations between IL-6 and any of the other biomarkers. Figure 3 provides an overview of the correlations between the inflammatory markers measured in the study population.

## 4. Discussion

To our knowledge, this is the first study in the literature in which beta-defensin 2, a novel biomarker of inflammation and intestinal dysbiosis, was measured in an infection-free population with overweight and obesity but without diabetes. Our findings indicate that beta-defensin 2 is positively correlated with presepsin, whereas we observed an interaction between various biomarkers that reflect distinct pathophysiological pathways, where inflammation acts as the connecting link between them.

Presepsin is the soluble form of the Gram-negative bacteria lipopolysaccharide (LPS) receptor and serves as a promoter of intracellular processes that regulate the immunological response to bacterial and fungal infections [14]. Although originally emerged as a biomarker of sepsis, recent data from our group suggest that it can accurately reflect metabolic dysregulation in infection-free subjects with type 1 and type 2 diabetes [15]. Furthermore, it is well correlated with conventional markers of glycemic control, such as continuous glucose-monitoring metrics [16]. It has been hypothesized that intestinal dysbiosis in people with metabolic disorders results in the penetration of increased LPS concentrations into the bloodstream, taking advantage of the vulnerability of the gut epithelial barrier [17]. The rise in LPS levels is reasonably accompanied by a respective increase in the amount of its linker, which is presepsin. We have recently shown that obesity is characterized by higher presepsin values compared to lean and overweight states [18]. The findings of the present study suggest that in overweight and obesity, beta-defensin 2 levels increase in parallel with presepsin, possibly as a counterregulatory mechanism against the harmful effects of metabolic endotoxemia.

In an animal model of diet-induced obesity, the oral administration of beta-defensin 2 enhanced not only intestinal epithelial barrier function but also metabolic indices, including glucose intolerance, hepatic fat content, and the number of liver lipid droplets [19]. In the same study, beta-defensin 2 improved the integrity of the gut epithelial barrier by upregulating tight junction and mucin expression. In agreement with our findings, these observations provide evidence for a close link between intestinal dysbiosis and metabolic dysregulation, although the translation of findings from animal studies into humans should be made with caution. In support of this perspective, among the various pro-inflammatory biomarkers we examined, beta-defensin 2 was significantly correlated only with presepsin, which could be attributed to the fact that intestinal microbiome imbalance constitutes the underlying pathophysiological disorder that connects these two markers.

Our findings are in line with previous studies that have evaluated hs-CRP in people with obesity. In a cohort of 3152 adults, Megawati et al. [20] recently reported that its levels are positively correlated not only with BMI values, but also with waist circumference, suggesting that hs-CRP is a reliable indicator of visceral adiposity. Adipose tissue functions as an endocrine organ by releasing cytokines, including tumor necrosis factor-α (TNF-α), interleukins, and various chemokines [21]. An excess of free fatty acids triggers pro-inflammatory pathways that promote cytokine production and stimulate the liver to synthesize CRP; transcription of the latter occurs predominantly in hepatocytes in response to increased cytokine levels, especially IL-6 [22]. Interestingly, pharmacological interventions that induce weight loss and alleviate cardiorenal risk in patients with obesity and established cardiovascular disease have been shown to parallelly improve hs-CRP levels, highlighting the crucial role of inflammation not only in the pathophysiology of the disease but also in its complications [23].

Our analysis suggested that the difference in hs-CRP levels between the different BMI categories became significant only when controls and participants with overweight were grouped together. The obvious reason behind this approach is to counteract the effects of the small sample size. However, there is an additional rationale. Studies have demonstrated that individuals with obesity exhibit significantly higher plasma concentrations of CRP and IL-6 compared to those with overweight, independent of age, sex, and metabolic comorbidities. Data from the National Health and Nutrition Examination Survey (NHANES) show that mean CRP levels in adults with obesity can exceed 5 mg/L, while in the population with overweight, values typically range between 2 and 3 mg/L, indicating a nearly two-fold increase in systemic inflammation in obesity [24]. This differential inflammatory status is attributable to both quantitative and qualitative changes in adipose tissue, including greater macrophage infiltration and a shift toward a pro-inflammatory M1 phenotype, as well as an enhanced production of adipokines such as leptin and resistin that further propagate inflammation [25]. These findings underscore that obesity confers a distinct and exacerbated inflammatory profile compared to the overweight state, with important implications for cardiometabolic risk and immune dysfunction. Thus, modest improvements in body weight and fat mass, particularly when translated into a regression from obesity to overweight, can significantly alleviate the inflammatory profile and, subsequently, improve health outcomes and mitigate the risk of complications in people with obesity.

The heterogeneity of obesity is increasingly recognized, with distinct phenotypes such as metabolically healthy obesity (MHO), metabolically abnormal obesity (MAO), metabolic obesity with normal weight (MONW), and sarcopenic obesity demonstrating varying levels of systemic inflammation. Studies have consistently shown that MHO individuals exhibit lower CRP levels compared to their MAO counterparts, despite similar degrees of adiposity, suggesting that systemic inflammation is more closely linked to metabolic dysfunction than to excess fat mass per se [26]. In MONW individuals, elevated CRP levels have been observed despite normal body weight, indicating that visceral adiposity and ectopic fat deposition, rather than BMI alone, contribute to inflammatory burden and cardiometabolic risk [27]. Sarcopenic obesity, characterized by the coexistence of excess adiposity and low skeletal muscle mass, is also associated with elevated CRP and other inflammatory markers, reflecting both adipose tissue-derived inflammation and muscle catabolism [28,29]. These findings underscore the complex interplay between body composition, metabolic status, and systemic inflammation in shaping obesity-related health outcomes.

Obesity is characterized by a chronic low-grade inflammatory state, in which altered secretion of adipokines and inflammatory cytokines plays a central role in metabolic dysregulation. Among the anti-inflammatory adipokines, vaspin, omentin, and irisin have emerged as important modulators of obesity-associated inflammation and insulin resistance. Vaspin, primarily expressed in visceral adipose tissue, has been shown to counteract the effects of pro-inflammatory cytokines such as TNF-α and IL-6, possibly through enhancement of insulin sensitivity and inhibition of NF-κB signaling pathways [30]. Similarly, omentin—secreted by visceral fat stromal cells—demonstrates anti-inflammatory properties, with inverse correlations reported between its circulating levels and markers such as CRP, IL-6, and TNF-α in individuals with obesity [31]. Irisin, a myokine also expressed in adipose tissue, appears to mediate anti-inflammatory effects by downregulating cytokine expression and promoting the browning of white adipose tissue, thereby reducing metabolic inflammation [32]. The interplay between these adipokines and inflammatory mediators highlights their potential role in the pathophysiology of obesity and related metabolic disorders.

We observed significant correlations between most of the inflammatory markers measured in the present study, which underscores the complex pathophysiological pathways that contribute to the genesis of adiposopathy. On the other hand, this fact highlights the potential value of the combined assessment of multiple biomarkers in the clinical evaluation of obesity. Emerging data suggest that such an approach may enhance predictive ability in terms of the risk of developing metabolic disorders, but also the probability of associated complications. Recent work by Hussain et al. [33] showed that a simultaneous evaluation of CRP, TNF-α, IL-6, interleukin-10, and adiponectin was effective in predicting the future risk of diabetes in a geriatric population. A “multimarker” approach has been suggested to facilitate the understanding of disease pathophysiology through multiple mechanisms, improve diagnostic and prognostic accuracy, and help develop prevention or management strategies for cardiometabolic disorders [34]. However, more research is needed before such an approach is widely implemented in daily practice.

The strengths of the present study lie in its novelty and the application of strict inclusion and exclusion criteria in an attempt to eliminate confounding effects on the findings and focus on the impact of obesity itself on the inflammatory state. In addition, the combined evaluation of various biomarkers provides a more detailed picture of the inflammatory milieu associated with obesity. On the other hand, our observations should be interpreted in light of important limitations, mainly the small sample size and the observational pilot nature of the study. Although the comparisons between individuals with obesity and those with overweight or normal weight did not reach conventional statistical significance, the evaluation of confidence intervals (CI) provides additional insight into the magnitude and uncertainty of group differences. Hs-CRP showed a modest separation in CI ranges (0.09–0.33 in obesity vs. 0.06–0.15 in controls/overweight) with a *p*-value approaching significance (*p* = 0.076), suggesting a possible trend toward higher systemic inflammation in obesity. However, after careful adjustment for several confounders, the difference in hs-CRP levels between the groups became significant (*p* = 0.044), confirming the robustness of our findings. In line with current methodological recommendations, the combined interpretation of *p*-values and CIs highlights the importance of considering both statistical significance and clinical relevance when evaluating group differences, particularly in studies with limited power or moderate sample sizes [35,36,37]. Furthermore, although BMI is currently considered the standard metric for the classification of obesity, it becomes increasingly evident that other anthropometric indices not evaluated in this study, such as waist circumference or waist-to-hip ratio, reflect the degree of adiposity more accurately and are better predictors of associated complications. Finally, we did not assess data on the dietary habits of participants that are known to interfere with the gut microbiome [38].

## 5. Conclusions

In conclusion, the findings of this pilot study suggest that obesity is characterized by an aggravation of systemic inflammation, as expressed by elevated levels of hs-CRP. We did not observe significant differences between the groups in the other inflammatory markers evaluated, including beta-defensin 2 which was the main focus of the study; however, it is possible that the small sample size limited the study’s ability to detect them. In addition, beta-defensin 2 levels correlate with presepsin concentrations in people with overweight and obesity who are free of infection and diabetes mellitus. These data reinforce the notion that gut dysbiosis is an important driver of the chronic pro-inflammatory environment that characterizes obesity. The correlations between the different biomarkers observed in this study suggest that inflammation represents the interface between pathogenetic pathways of different origin that culminate in but also arise from increased adiposity. Future studies with a large sample size, adequate statistical power, and a prospective design are needed to confirm or refute our findings. Longitudinal studies should evaluate the clinical utility of combined biomarker use to predict the risk of developing obesity and its complications and explore whether interactions between the various inflammatory markers represent genuine pathophysiological alterations and denote causality. Additionally, the possible role of gut dysbiosis markers in the establishment of different obesity phenotypes, which could allow for a more appropriate treatment selection and predict the response to anti-obesity medications, is an area for future research.

## Figures and Tables

**Table 1:** Inflammatory markers in the study population.

Biomarker	Group	Mean	SD	Median	Minimum	Maximum	95% CI for the Median	*p*-Value
Presepsin (ng/mL)	Control or Overweight	3.97	3.98	2.56	0.82	22.30	1.79–3.00	0.175
	Obesity	5.61	5.00	3.18	1.24	16.07	2.10–6.54	
Beta-defensin 2 (pg/mL)	Control or Overweight	181.99	122.94	152.95	136.45	919.50	146.55–162.55	0.936
	Obesity	165.00	30.52	157.40	136.70	267.70	146.17–165.93	
Ferritin (pg/mL)	Control or Overweight	85.28	69.31	71.34	6.71	320.80	51.10–92.64	0.237
	Obesity	113.38	85.74	90.39	8.87	329.10	49.70–153.55	
IL-6 (pg/mL)	Control or Overweight	3.38	3.71	2.00	1.50	22.51	1.50–2.68	0.341
	Obesity	3.76	2.98	2.21	1.50	11.11	1.60–4.28	
hs-CRP (mg/dL)	Control or Overweight	0.20	0.27	0.10	0.00	1.24	0.06–0.15	0.076
	Obesity	0.36	0.42	0.13	0.01	1.37	0.09–0.33	

SD: standard deviation; IL-6: interleukin 6; hs-CRP: high sensitivity C-reactive protein; CI: confidence interval.

## Data Availability

The data presented in the study are available on request from the corresponding author. The data are not publicly available due to privacy restrictions of the Greek National Health System.

## Supplementary Materials

The following supporting information can be downloaded at: https://www.mdpi.com/article/10.3390/biomedicines13081800/s1, Table S1: Demographic, anthropometrical, and laboratory characteristics of the study cohort categorized by weight groups.
