# Supplementary material for: Circulating Beta-Defensin 2 Levels Correlate with Conventional Inflammatory Markers in Infection-Free Individuals with Overweight and Obesity: An Exploratory Study
Source: Biomedicines. 2025 Jul 23;13(8):1800. doi: 10.3390/biomedicines13081800 (PMC12383832; doi:10.3390/biomedicines13081800)
Supplement: Supplementary file 1 [file biomedicines-13-01800-s001.zip › biomedicines-3726823-supplementary.pdf]

Supplementary Table S1. Demographic, anthropometrical, and laboratory characteristics of the study cohort categorized by weight groups

	**Group**									
	**Control (n = 20)**			**Overweight (n = 34)**			**Obesity (n = 27)**			***p*-Value**
	**Mean**	**SD**	**Median**	**Mean**	**SD**	**Median**	**Mean**	**SD**	**Median**	
Age (years)	43.45	11.98	46	49.88	13.01	50	47.44	10.09	50	0.542
Weight (kg)	64.75	8.88	64	78.50	11.22	75	98.07	21.79	90	<0.01
Height (m)	1.69	0.09	1.67	1.69	0.12	1.67	1.67	0.10	1.67	0.752
BMI (kg/m^2)	22.53	1.19	22.9	27.45	1.31	27.5	35.18	5.86	33.4	<0.01
Duration of supplementation (months)	6.07	10.80	0.5	1.90	5.74	0.0	1.44	2.98	0.0	0.324
25(OH)D (ng/mL)	28.19	9.21	29.2	25.63	7.02	25.3	24.24	5.28	23.2	0.179

BMI: body mass index; SD: standard deviation; 25(OH)D: 25-hydroxy-vitamin D
